# Numerical study of lateral coolant jet on heat reduction over nose cone with double-aerodome at hypersonic flow

**DOI:** 10.1038/s41598-022-22061-y

**Published:** 2022-11-27

**Authors:** Mehdi Ghanbari, Soroush Maddah, Javad Alinejad

**Affiliations:** Department of Mechanical Engineering, Sari Branch, Islamic Azad University, Sari, Iran

**Keywords:** Aerospace engineering, Mechanical engineering

## Abstract

One of the main challenges in designing a supersonic forebody is thermal protection. The application of the mechanical spike mounted at the nose considerably decreases the heat load on the main body. In this investigation, the hybrid technique of mechanical spike and coolant injection are examined to reduce the thermal load on the nose cone in the supersonic air stream. A three-dimensional model of a double aerodisked spike with different cooling systems is provided to find the efficient cooling injection system for reducing the heat load on the nose cone. Computational studies have been done on investigating a cooling mechanism in the proposed injection systems. This study has tried to present valuable information on flow features and shock interaction nearby the nose. The influence of different coolant gas on the thermal performance of the proposed configurations is comprehensively explained. Our results indicate that the cooling performance of single carbon dioxide is 85% more than helium jet in lateral injection. According to our findings, the cooling performance of lateral multi-jets is 90% more than opposing ones.

## Introduction

The importance of hypersonic vehicles is undeniable for humans and scientists who believe the future is a world of fast transportation systems. Indeed, high-speed flight not only enable humans to quick civil flight but also help human to move beyond the earth via shuttle^1–3^. These are the main concepts for the scientists who have tried to improve the efficiency of the current high-velocity vehicles. Besides, the concept of high-speed flight has always been supported by the government which wants to improve the military application^4,5^. These benefits encourage aerospace scientists to pay more attention to developing existing high-velocity vehicles. It is also found that progress in space science has lots of advantages for human life since the new knowledge achieved in this field could be used for other science and applications^6,7^. It was initially assumed that the main challenge for the development of the subsonic to supersonic vehicles is the power and efficiency of the engine for the production of the required thrust and lift force in a low-density domain. In fact, the strong shock in the tip of the nose of the high-speed vehicles has diverse disadvantageous for the performance of hypersonic vehicles^8,9^. Indeed, it is found that the heat and shock production in the tip of the high-speed vehicles has induces lots of problems such as noise radar transmission and burning of the nose due to excessive heat production. This phenomenon known as aerodynamic heating has been widely focused on by aerospace and physic scientists. The nature of this phenomenon has numerous unknown aspects which require high attention^10–12^.

The flow analyses of the hypersonic vehicles are widely done to disclose the effective factors and parameters associated with aerodynamic heating^13–16^. Although these studies have presented significant data about the mechanism of the aerodynamic heating around the nose of the cone, there are still some challenges i.e. laminar to turbulence transition, heat reduction technique, and turbulence simulations^17^. Due to production of the excessive heat nearby the nose, the control of the unadorned aeroheating has become a vital challenge for the design of hypersonic vehicles. Meanwhile, drag force is another factor that should be considered for the design of an efficient forebody for the high-speed vehicle^18–20^. Hence, there are some configurations that are developed for the reduction of aerodynamic heating and drag force. Three main devices have been introduced for the control of aerodynamic heating: mechanical, fluidic, and thermal devices^21,22^. These techniques have their own pros and cons and these are extensively studied in previously published papers. Among these devices, the mechanical spike is the simplest and most practical technique that is used in real models^23,24^. Aerodisk spike is known as the most efficient version of a mechanical device for the reduction of heat load on the main body. Other techniques are developed on a lab scale and investigated in academic studies^25–27^.

**Figure 1:**
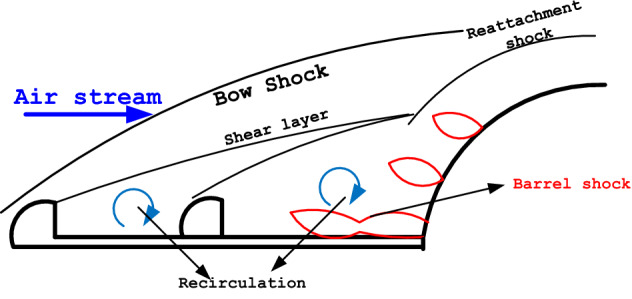
Schematic of multi-jets system for cooling of nose cone with double aerodisk.

Recently, hybrid devices have also become popular since these combined techniques reduce the disadvantage of the main concept and improve the overall efficiency of the forebody at high-speed flight^2,28,29^. The mechanical device of the spike has lots of advantages Since the spike is the most convenient device for real applications, scholars have tried to combine this method as the primary device with the fluidic and thermal approach^30,31^. A fluidic jet has acceptable efficiency for local cooling and could reduce the temperature of the main body^32–35^. Thus, we have tried to investigate a new configuration that includes aerodisked spike and fluid device. As demonstrated in Fig. 1, the injection of both single and multi-jets nearby the nose cone with a double aerodisked spike at a hypersonic free stream. The main expected advantage of this model is the heat reduction of air nearby the main body while the drag of the main body is managed via spike with mounted aerodisked. This separation between the heat and drag force mechanism improves the overall performance of the whole model in different conditions.

In this paper, computational fluid dynamics is used to study the hybrid technique of mechanical and fluidic devices simultaneously on the heat and drag reduction mechanism of the nose cone at the hypersonic free stream. Several coolant injection systems are examined on the nose cone with double aerodisk at supersonic flow. Shock interactions and coolant effects on the heat transfer of the nose cone are extensively explained. This study also reveals the impacts of coolant gas (Helium and Carbon dioxide) on the heat rate of the main body. A three-dimensional model is produced to consider the main physic of the main flow and coolant jet. Various jet locations are also compared and the efficient jet configuration is introduced in this work.

## Governing equations

To study the compressible flow around the nose cone, RANS equations are coupled with the SST turbulence model to simulate the three-dimensional supersonic flow around the nose cone with a double aerodisk^36–39^. Since inert gases of helium and carbon dioxide are selected for cooling the nose cone, a species transport equation is also required for the simulation of the proposed configuration. OpenFOAM^40^ free code is used for computational simulation of the double aerodisked spike with coolant injections. The second-order upwind scheme of Roe is used to discretize the convective terms. The mixing law is used for the calculation of the heat transfer coefficient in the vicinity of the main blunt-body^41–43^.

**Figure 2:**
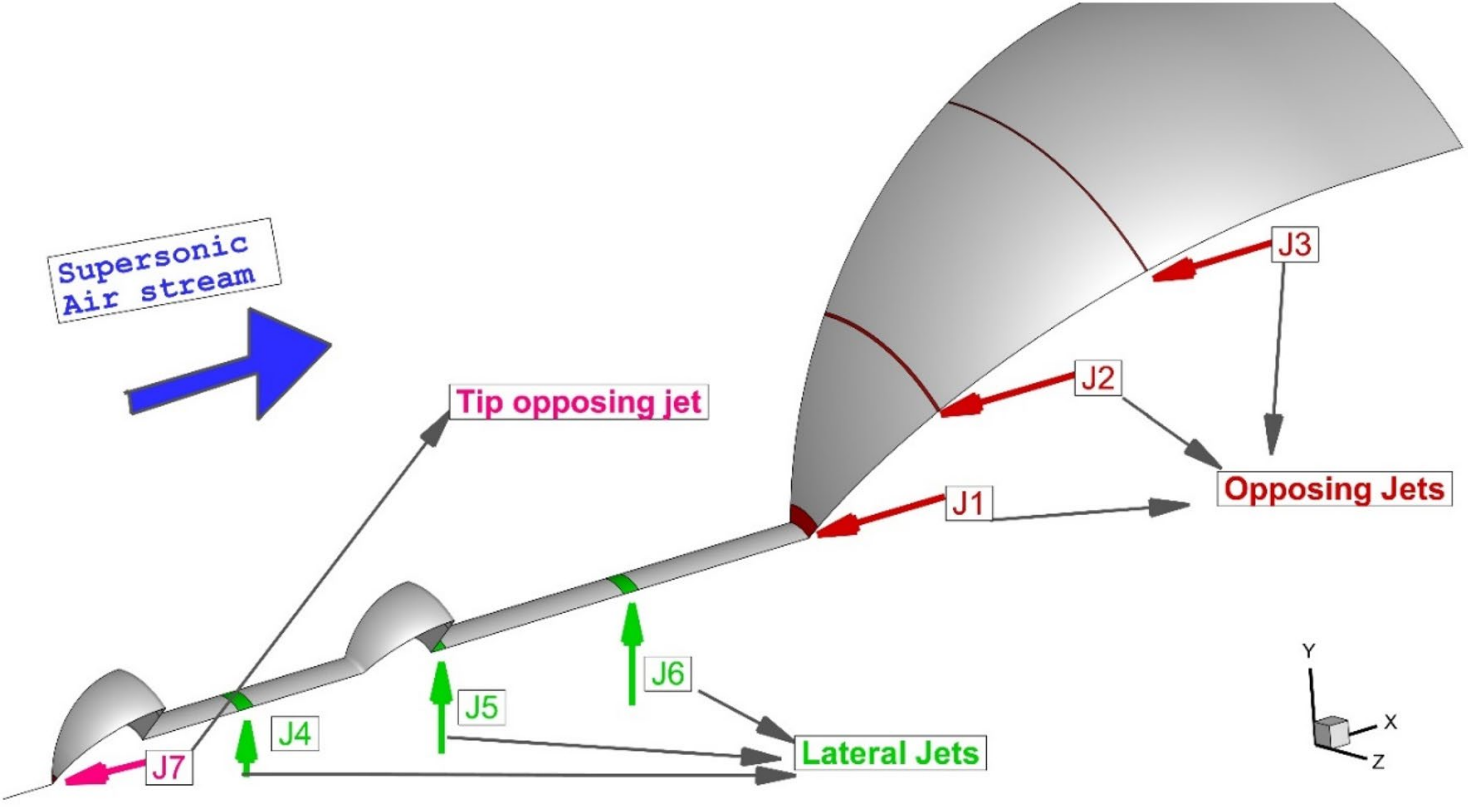
Injection system for cooling of the nose cone with double aerodisked spike.

In the selected model (Fig. 2), the diameter of the nose cone is 50 mm. The sonic nozzle has a diameter of 4 mm. The length of the spike is equal to the diameter of the nose cone. The supersonic air flow with Mach and static pressure of 5 and 2550 Pa, respectively, at a static temperature of 221 K is applied via pressure far-field boundary condition. Helium and carbon dioxide are injected from different locations at sonic velocity with a total temperature of 300 K. The jet total pressure is about 10% of the total pressure of the free stream. The constant temperature is applied to the body wall. Six jet locations on the nose and spike are investigated in our study. The angles of the injector on the nose cone are 0, 30, and 60°.

A grid study is also performed for the correct simulation of the compressible flow around the nose cone with a double aerodisk. Since the shock is produced nearby the main body and spike, the production of the grid in these regions is done via specific considerations. The size of the grid nearby the nose is lower while the aspect ratio and cell skewness of produced grids are kept in a standard range to avoid divergence. The grid study is also examined with four different grid sizes on two lines (θ = 30 and 60) on the main body as presented in Table 1. Four different grids are examined in this study for the grid independency analysis. It is found that the fine grid is a reasonable grid for our model. 72 h are computational time while our residual become less than 10e−4.

**Table 1:** Grid details.

Model	Grid number	Average Stanton numb. on blunt cone (θ = 30)	Average Stanton on numb. blunt cone (θ = 60)
Coarse grid	680,000	0.00212	0.00618
Normal grid	960,000	0.00245	0.00637
Fine grid	1,320,000	0.00251	0.00651
Very fine grid	1,680,000	0.00253	0.00653

## Results and discussion

### Validation

**Figure 3:**
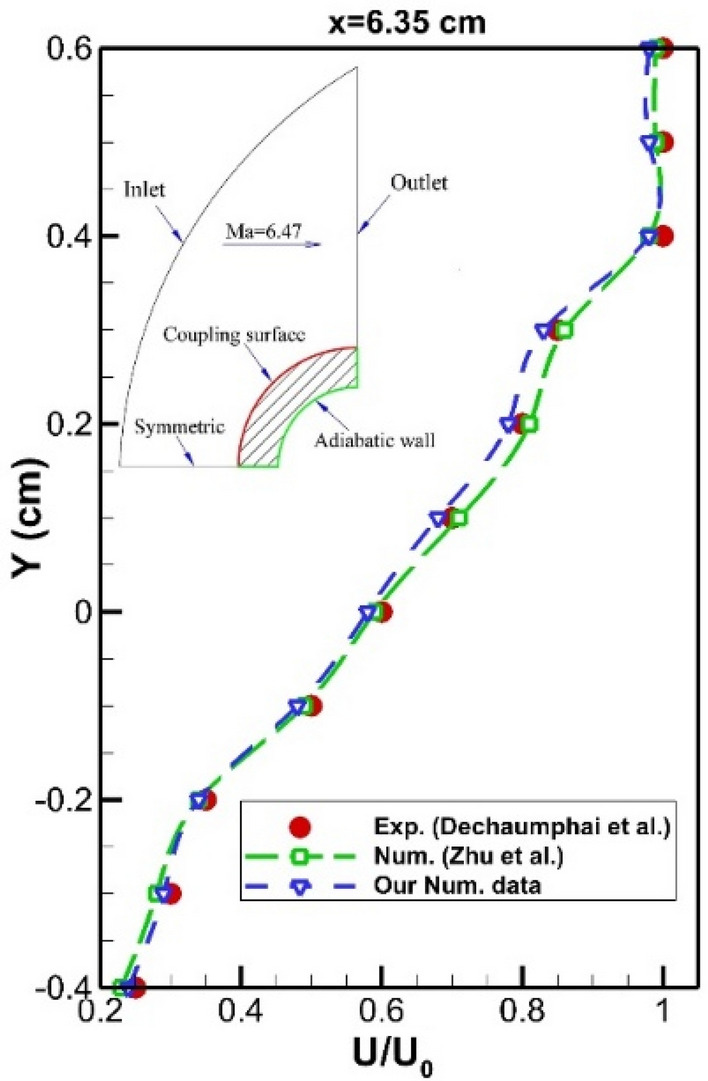
Validation of velocity profile^45^.

To certify the proposed model and obtained results, we must initially compare our results with experimental data. Since experimental data of the simple cone^44^, we initially simulated the flow characteristic around the nose cone without a spike. Figure 3 demonstrated the velocity profile at a specific location and pressure distribution on the main body for the simple model. The numerical data of Zhu et al.^39^ is also presented in these figures for the verification of our results. According to our comparison, the deviation of our numerical simulation with experimental data and other computational studies is within an acceptable range^46–50^. Finite volume technique is extensively used for different engineering problems^51–54^.

### Flow analysis

In this study, the flow study of the single jet at different locations (3 lateral jets and 3 opposing jets) is fully investigated. 3-D results are also presented to clarify the main impacts of the single jet on flow features and shock interactions nearby the nose body. Then, dual jets (one lateral jet + one opposing jet) are investigated for helium jets. Finally, the existence of three either lateral or opposing jets is fully explored for both helium and carbon dioxide jets. The influence of these jet configurations on the heat load reduction of the main body would be disclosed.

**Figure 4:**
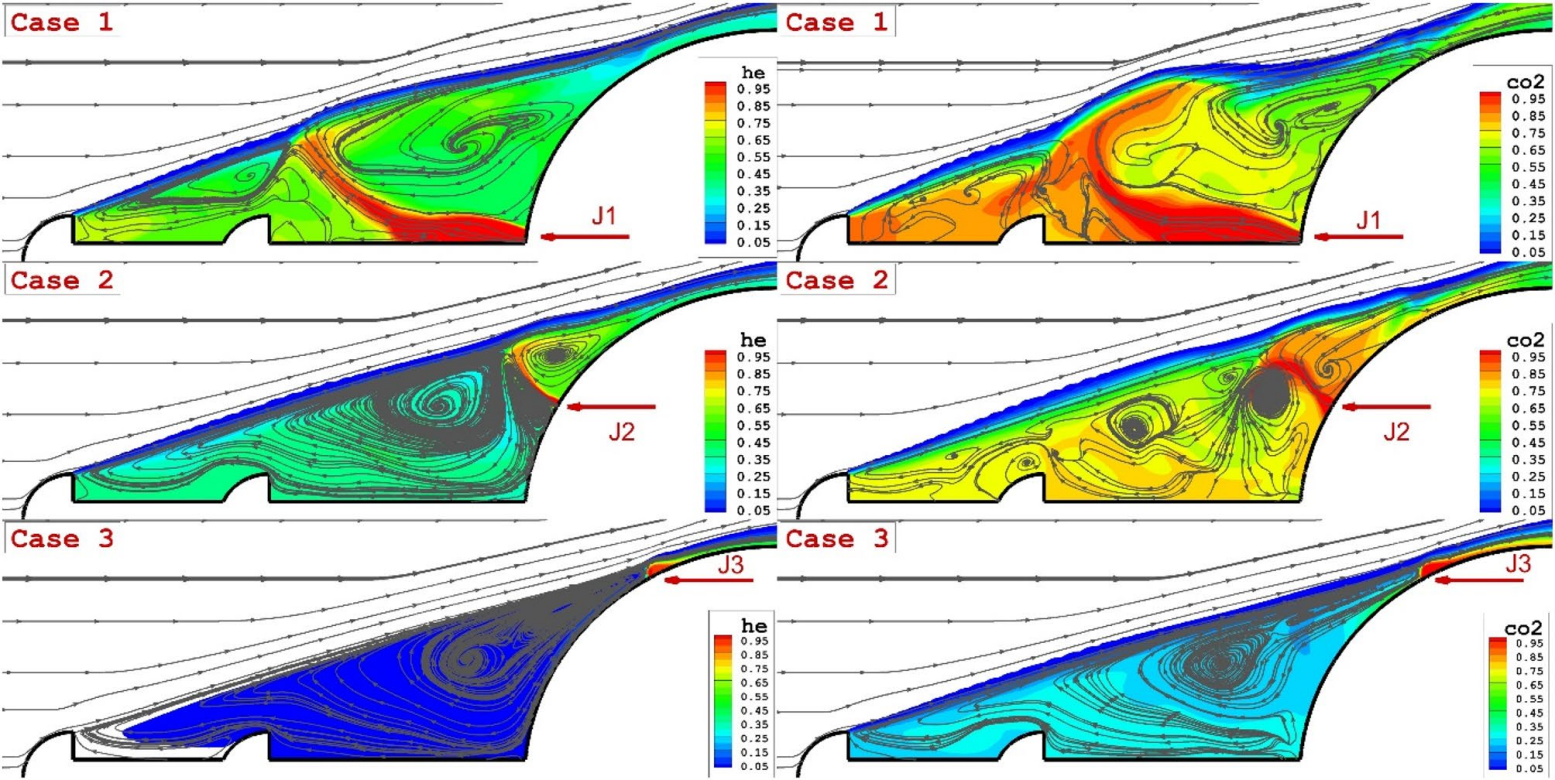
Comparison of helium (left side) and CO_2_ (right side) opposing jet for different location on the main body.

Figure 4 demonstrates the flow feature around the spiked nose cone with double aerodisks in presence of a single opposing coolant jet at the hypersonic free stream. The contour is presented on the plane in the mid-section of the domain. A comparison of the Helium and carbon dioxide indicate that the concentration of the carbon dioxide is higher nearby the nose cone and this is favorable for the cooling system. A comparison of the streamlines shows that the structure of the circulation is preserved by injection of the helium jet since the Helium jet tends to penetrate straightly rather than radially. As the jet injection moves up, the reattachment point moves downstream.

**Figure 5:**
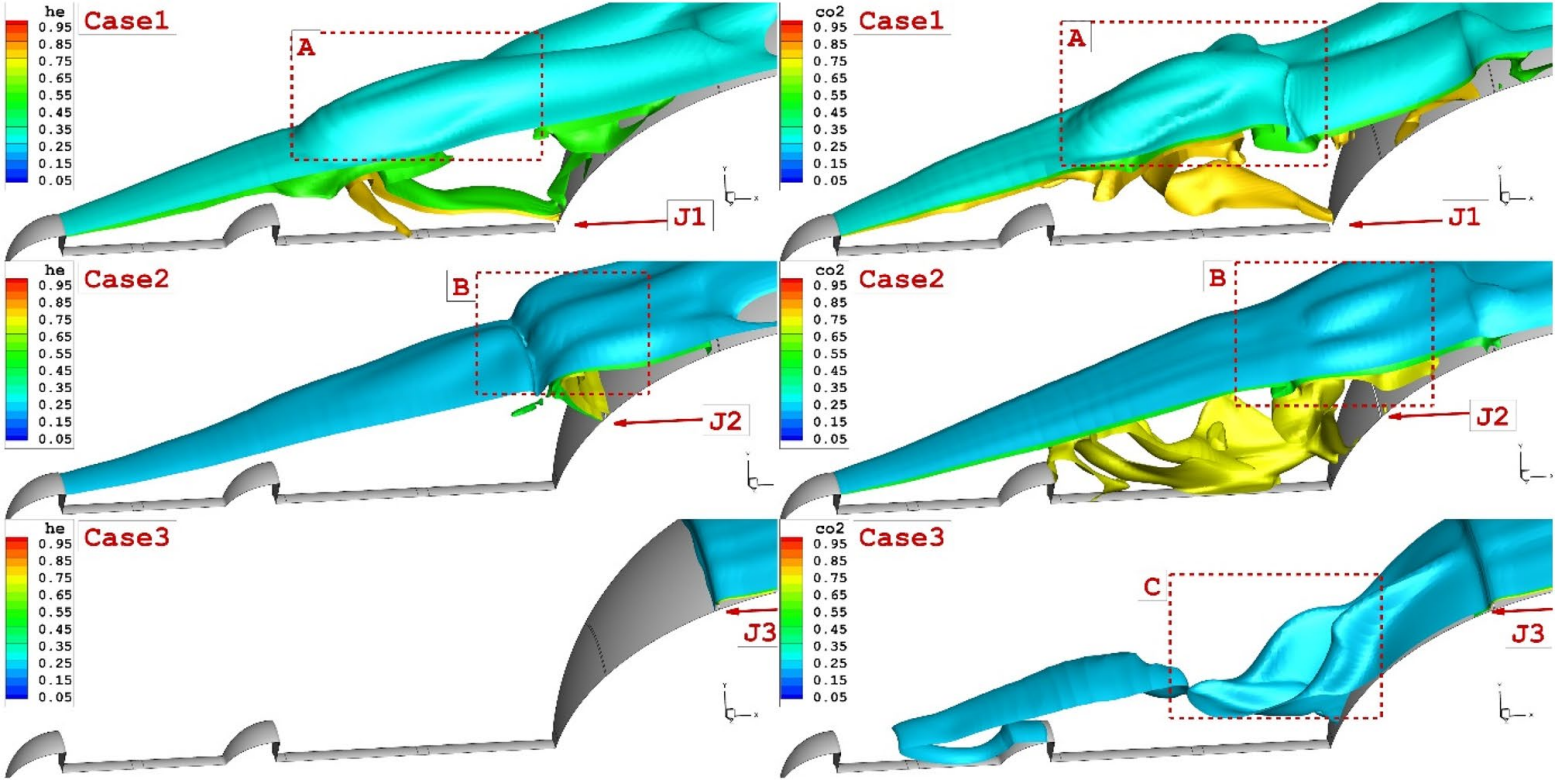
Effects of 3-D shock interaction of helium and CO_2_ opposing jet in different location on the penetration of the coolant jets.

Figure 5 illustrates the mass contour of the single opposing jet of helium and carbon dioxide. Our results indicate that the interaction power of the carbon dioxide coolant jet is more than the helium one. In fact, the high concentration of the carbon dioxide jet in the recirculation region provides a high-pressure region which increases the angle of the shear layer. The three-dimensional feature of the coolant jet indicates the influence of the shock interactions in the vicinity of the jet and shear layer (Fig. 5). The fluctuation of the coolant layer demonstrates the impacts of different jet locations on the flow feature nearby the shoulder of the nose cone.

**Figure 6:**
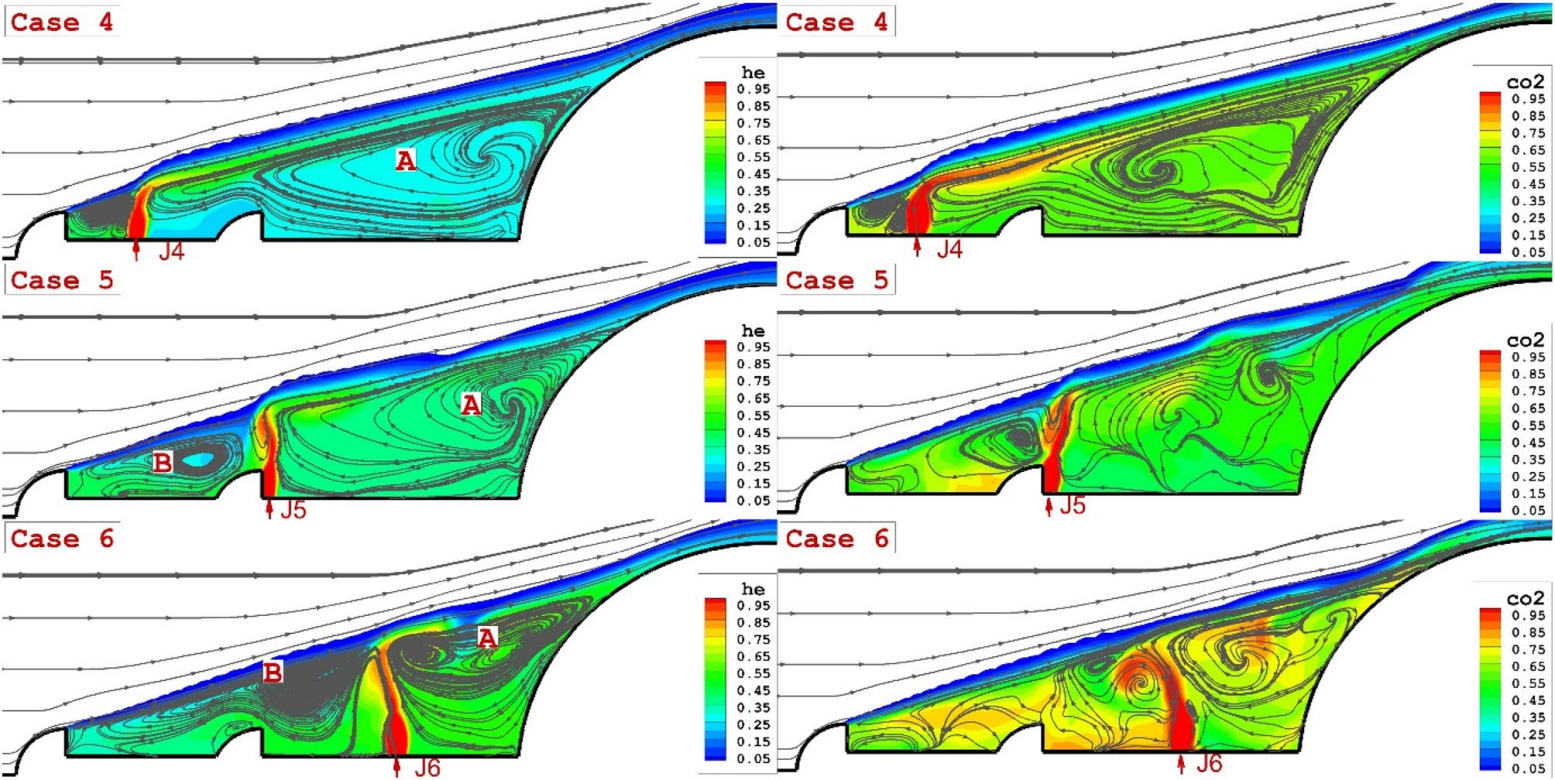
Comparison of helium (left side) and CO_2_ (right side) lateral jet for different location on the main body.

The impact of the single lateral injections of coolant gas on the flow stream and coolant distribution is demonstrated in Fig. 6. It is found that the injection of the lateral jet increases the number of recirculation nearby the spike. A comparison of the helium and carbon dioxide jet indicates that the helium jet preserves the circulation structure as it is observed in the opposing jet. The existence of a second aerodrome avoids the separation of the circulation on the spiked rod.

**Figure 7:**
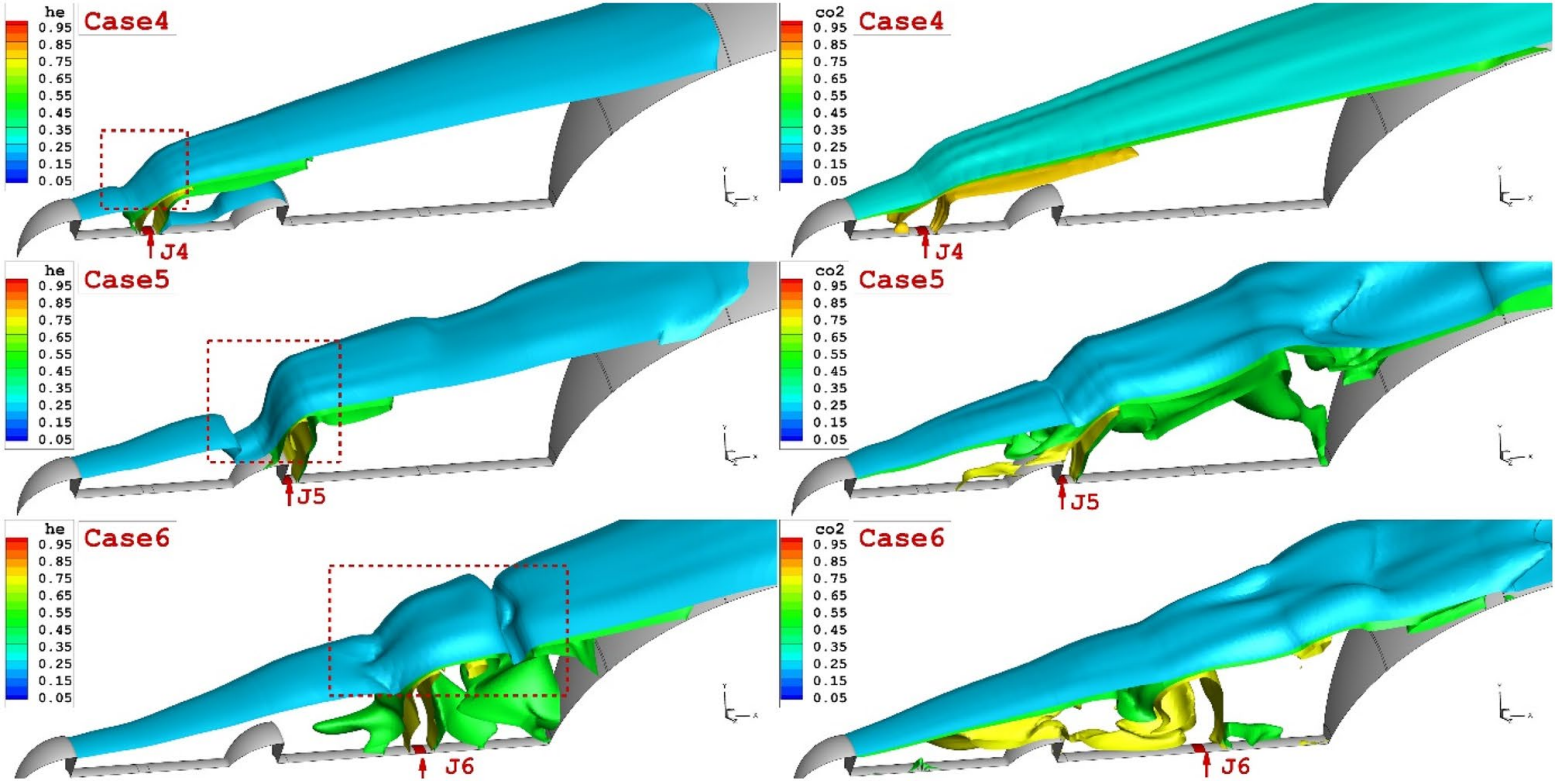
3-D feature of the coolant jets (helium and CO_2_) on the structure of the flow stream nearby the main body.

Figure 7 demonstrates the jet interactions on the specific plane located on the mid-angle of the domain. The impacts of the lateral helium and carbon dioxide for different jet locations are illustrated. Our comparison indicates that the shock deflection is more clear when the jet location is close to the tip of the first aerodrome (J4). In fact, the gap of the shear layer with bow shock decreases when the coolant jet is injected into the vicinity of the first aerodome. A comparison of helium and carbon dioxide also confirms that the higher penetration of the helium jet is more effective on the deflection of the shear layer. Figure 7 also confirms the role of the helium jet on the flow feature around the forebody in comparison with carbon dioxide.

**Figure 8:**
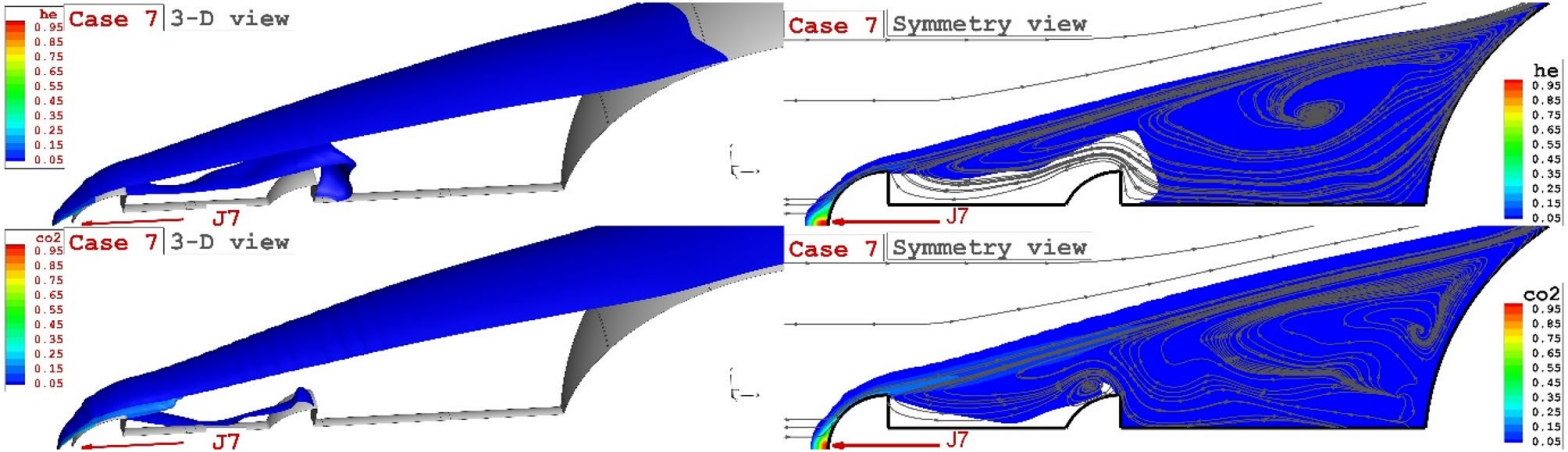
Comparison of the helium.

Figure 8 demonstrates the flow feature of the helium (upper figure) and carbon dioxide jet (lower figure) injected from the tip of the aerodome. Our results indicate that the carbon dioxide jet efficiently disperses the incoming air stream and this deflects the bow shock with a higher angle. Consequently, the attachment point on the main body does not occur on the main body, and the heating of supersonic flow is decreased.

### Heat transfer

**Figure 9:**
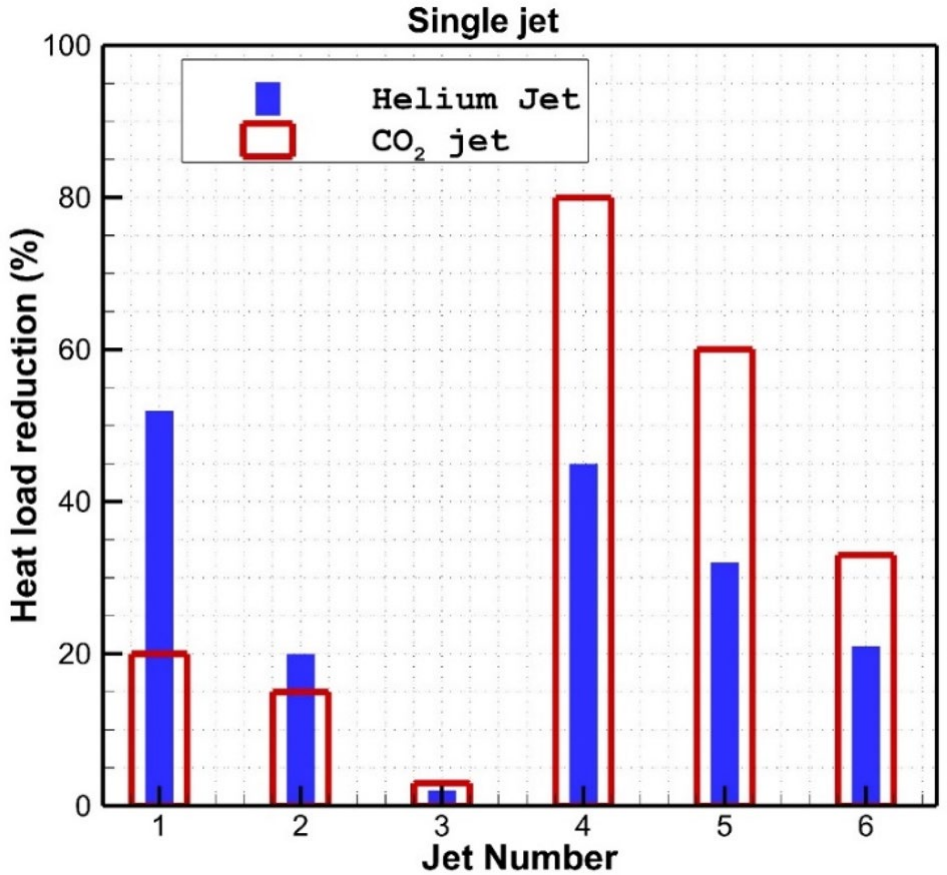
Comparison of the heat load reduction of single coolant jet on the main body for different jet configurations.

Figure 9 compares the heat reduction performance of these jet configurations for both helium and carbon dioxide on the main body. As introduced in Fig. 2, jet numbers 1, 2, and 3 are opposing jets while the jet numbers 4, 5 and 6 are lateral jets. Our results indicate that lateral jet injection is more efficient in heat reduction than opposing jets when carbon dioxide is selected as a coolant. The impact of the helium opposing jet decreases when the jet becomes close to the shoulder of the main blunt body.

## Conclusion

This study has performed several computational investigations to investigate the impacts of cooling injections on the thermal performance of the spiked nose cone with double aerodomes. The injection of single and multiple coolant jets is fully investigated at the supersonic free stream of M = 5. A 3-D model is developed for our investigations to observe real aspects of flow features near the nose cone. Injection of carbon dioxide and helium are compared on the proposed models. Our investigation indicates that, in opposing configuration, helium jet is more efficient for heat reduction in both single and multi-jets. Our results show that the cooling performance of single carbon dioxide is 85% more than helium jet in lateral injection. It is also observed that the helium jet is more efficient than carbon dioxide for the cooling system of the opposing jet.

## Data Availability

All data generated or analysed during this study are included in this published article.
